# Screening of Potential Anti-Thrombotic Ingredients from *Salvia miltiorrhiza* in Zebrafish and by Molecular Docking

**DOI:** 10.3390/molecules26226807

**Published:** 2021-11-11

**Authors:** Huilan Tang, Ningyi Qin, Chang Rao, Jiahui Zhu, Haiqiang Wang, Guang Hu

**Affiliations:** 1 School of Pharmacy and Bioengineering, Chongqing University of Technology, Chongqing 400054, China; BellaT@2019.cqut.edu.cn (H.T.); raochang2021@hotmail.com (C.R.); zhujh@2020.cqut.edu.cn (J.Z.); whq723366140@hotmail.com (H.W.); 2 Chongqing Pharmaceutical Group Huamosheng Pharmaceutical Science & Technology Co., Ltd., Chongqing 400050, China; vivianqny@163.com; 3 Chongqing Key Laboratory of Medicinal Chemistry & Molecular Pharmacology, Chongqing University of Technology, Chongqing 400054, China

**Keywords:** zebrafish, molecular docking, salvianolic acid B, rosmarinic acid, lithospermic acid, luteolin-7-O-β-d-glucoside, anti-thrombotic effects

## Abstract

Background: Danshen (DS), the dry root of *Salvia miltiorrhiza* Bge., has been used in traditional Chinese medicine (TCM) for many years to promote blood circulation and to inhibit thrombosis. However, the active ingredients responsible for the anti-thrombotic effect and the underlying mechanisms are yet to be fully elucidated. Methods: Molecular docking was used to predict the active ingredients in DS and their potential targets by calculating the scores of docking between DS ingredients and thrombosis-related proteins. Then, a chemical-induced zebrafish thrombosis model was applied to confirm their anti-thrombotic effects. Result: The molecular docking results indicated that compared to the control ligand, higher docking scores were observed for several compounds in DS, among which salvianolic acid B (SAB), lithospermic acid (LA), rosmarinic acid (MA), and luteolin-7-O-β-d-glucoside (LG) could attenuate zebrafish caudal vein thrombosis and recover the decrease in heart red blood cells (RBCs) in a dose-dependent manner. Conclusions: Our study showed that it is possible to screen the potential active components in natural products by combining the molecular docking method and zebrafish in vivo model.

## 1. Introduction

Thrombosis is characterized by intravascular thrombus formation and vessel occlusion, which may result in myocardial infarction, ischemia, and stroke, and it can be especially dangerous if it blocks blood flow to organs or tissues [1]. Thrombus formation in blood vessels is usually associated with systemic disorders such as hypercoagulability [2]. As the leading cause of death worldwide [3,4], thrombosis, which plays an important role in cardiovascular disease, seriously threatens human health and life [5,6]. Therefore, new drug development in thrombosis treatment is in great demand.

Traditional Chinese medicine (TCM) has been widely applied in clinical therapy in China and other countries [7,8,9] for a long time. According to the TCM theory, thrombosis belongs to the syndrome of blood stasis. Many herbal formulae and extracts are used to treat blood stasis syndrome (BSS) via promoting blood circulation, inhibiting platelet aggregation, and promoting the hemostasis release reaction [10,11]. Danshen (DS), the dry root of *Salvia miltiorrhiza* Bge. which can also be used in food and healthy supplement [12,13], has attracted increasing attention as a treatment option for cardiovascular and cerebrovascular diseases [13,14]. DS, which contains more than 200 chemical constituents including salvianolic acid B (SAB), danshensu (DSS), lithospermic acid (LA), rosmarinic acid (MA) [15], and luteolin-7-O-β-d-glucoside (LG), is a commonly used medicine for “invigorating’’ the blood and reducing blood clotting [16]. For example, many studies have shown that DS can inhibit platelet aggregation and adhesion, improve blood microcirculation, and reduce thrombus formation [17]. Therefore, DS has been extensively used to cure cardiovascular and cerebrovascular diseases, inflammatory diseases, and neurodegeneration diseases [15]. Furthermore, it has been demonstrated that the extract of the herb has effects against hemostasis as well as eliminating blood stasis. However, the active ingredients responsible for the anti-thrombotic effect in DS and their target proteins are yet to be fully elucidated.

Virtual screening based on molecular docking is a computational technique used in drug discovery research. It can deal with the quick search of large molecular libraries to identify the structures that are most likely to bind the drug target, which is typically a protein receptor or enzyme [18]. In recent years, molecular docking, which plays an important role in drug screening and design [19], is one of the most frequently and effectively used methods to predict the optimal binding mode (binding sites and binding poses) and affinity between a small molecule drug candidate (ligand) and a target protein (receptor) [20,21,22]. Several compounds including lithospermate B, salvianolic acid C, lithospermate acid B, magnesium lithospermate B, danshensuan B, salvianolic Acid B, monomethyl lithospermate, and oleoyl neocryptotanshinone in DS have been identified as ingredients with potential anti-thrombotic activity with this approach [18].

Zebrafish (*Danio rerio*) has been used in the past few years to identify novel factors of hemostasis and thrombosis, and to analyze their functions in greater detail [23,24,25,26]. Compared to the conventional mammal in vivo models, which are usually laborious, costly, and time consuming [27], the zebrafish model organism has a large number of advantages including high fecundity, small size, rapid development, and rapid generation time [28]. In fact, zebrafish is considered to be especially appropriate for the research of angiocardiopathy than other models because the endogenous blood circulation can be dynamically observed without invasive methods [16], and there is remarkable similarity in the cardiovascular system between zebrafish and humans [29,30]. Furthermore, with the capability of percutaneous oxygen absorption, zebrafish embryos could survive for a long time without functional blood circulation, during which the investigation of anti-thrombotic agents could be done [31].

In the present study, virtual screenings based on molecular docking between components in DS and thrombosis-related protein targets were performed. Potential active ingredients and possible protein targets were predicted according to the docking scores. Then, candidates of active compounds in DS with potential anti-thrombotic effect were treated to chemical-induced thrombosis zebrafish to validate their ability of promoting blood circulation and eliminating blood stasis.

## 2. Results and Discussion

### 2.1. Analysis of Molecular Docking

**Figure 1:**
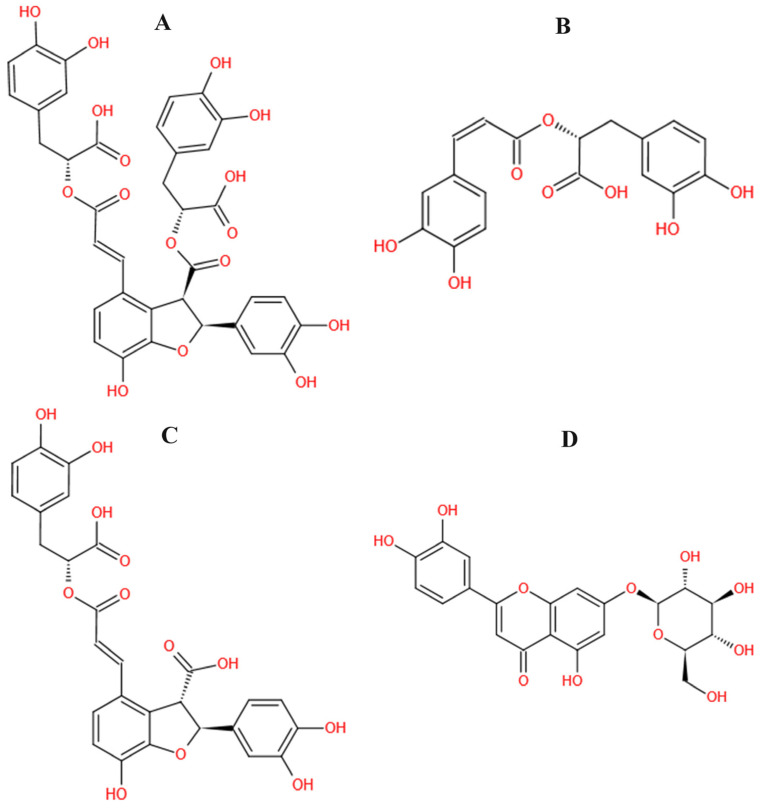
The structure of salvianolic acid B (SAB), rosmarinic acid (MA), lithospermic acid (LA), and luteolin-7-O-β-d-glucoside (LG). (**A**–**D**) indicated as SAB, MA, LA, and LG, respectively.

The structure information of 202 compounds in DS were obtained from the Traditional Chinese Medicine Systems Pharmacology Database and Analysis Platform (TCMSP) (https://old.tcmsp-e.com/tcmsp.php, accessed on 4 November 2021). The relevant target proteins of thrombosis were collected in the Therapeutic Target Database (TTD) (http://db.idrblab.net/ttd/, accessed on 4 November 2021), and 31 target proteins were obtained, including a successful target, clinical trial target, discontinued target, and literature-reported target. Then, the corresponding X-ray crystallographic structures with higher resolution were searched through the UniProt (https://www.uniprot.org, accessed on 4 November 2021) database, and 25 crystal structures were selected from the Protein Data Bank (PDB) (http://www.rcsb.org/, accessed on 4 November 2021) database for subsequent experiments. Finally, 202 active ingredients in DS were docked with 25 target proteins respectively, and the results are shown in Table 1 and Table 2. On one hand, a thrombosis-related target protein could combine well with multiple ligands, e.g., antithrombin-III (ATIII, PDB ID:1R1L) could bind to 160 ingredients in DS; on the other hand, one compound in DS would bind to 10–15 thrombosis-related targets. According to the docking result, many small molecules in DS could bind to the same proteins including 1R1L, 5AFY, 4C2A, 6TS4, 3QX3, 3T3M, 2JKH, and 5AFN. Among all the compounds that could combine with multiple thrombosis-related targets in Table 2, SAB, MA, LA, and LG (structures shown in Figure 1), which were characteristic compounds of DS and can be commercially obtained with high purity, were selected for further in vivo pharmacological tests. The docking scores of SAB, MA, LA, and LG are shown in Table 3.

**Figure 2:**
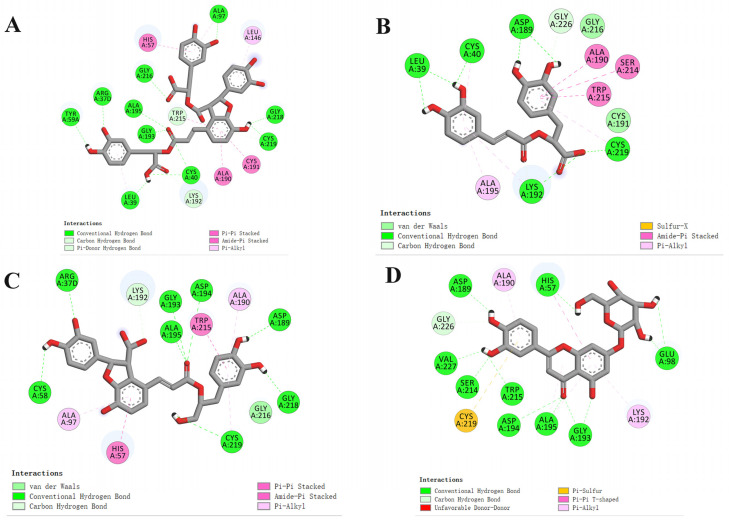
Docking results of the compounds SAB (**A**), MA (**B**), LA(**C**), and LG (**D**) with the coagulation factor XI (F11) (PDB ID: 6TS4).

To further explore the binding mode between the receptor and ligands, SAB, MA, LA, and LG were selected as active compounds for the molecular docking study. The interaction diagrams of the best-docked conformations are shown in Figure 2. The hydrogen bonds and amino acid residues of the interaction between coagulation factor XI (F11) (PDB ID: 6TS4) and the compounds are presented in Table 4. Hydrogen bonds play an important role in the binding of ligands to F11 that exist between SAB and TYR59A, ARG37D, GLY193, ALA195, GLY216, ALA97, GLY218, CYS219, CYS40, LEU39; MA and LEU39, CYS40, ASP189, CYS219, LYS192; LA and CYS58, ARG37D, ALA195, GLY193, ASP194, ASP189, GLY218, CYS219; as well as LG and ASP189, HIS57, GLU98, GLY193, ALA195, ASP194, TRP215, SER214, VAL227. Particularly, ALA195, GLY193, GLY216, CYS219, ASP189, and LYS192 are the most frequent amino acid residues, which may be the important residues for the interactions between ligands and F11. Moreover, the compounds containing hydroxyl and carbonyl, especially phenolic hydroxyl groups, are vital for hydrogen bond formation with F11. These results indicated that these compounds may be the potential active ingredients in DS with an anti-thrombotic effect.

### 2.2. Assessment of the Anti-Thrombotic Effect of SAB, MA, LA, and LG

**Figure 3:**
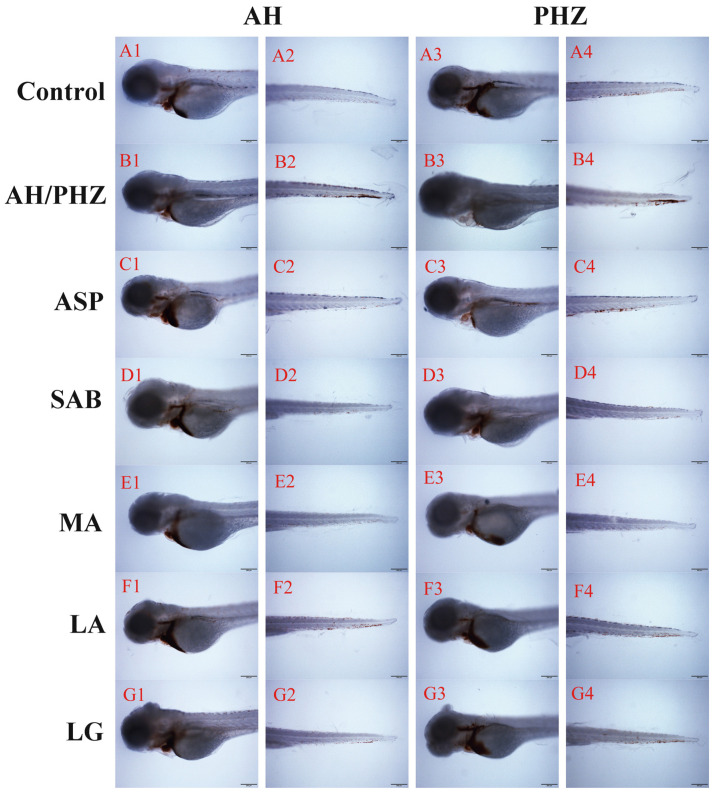
The RBC staining intensity of heart or caudal vein in zebrafish thrombus treated with different DS compounds. Rows (**A**–**G**) indicated the control, AH/PHZ, ASP, SAB, MA, LA, and LG group, respectively. Chemical inducers in columns 1 and 2 are AH, while PHZ is used for columns 3 and 4.

Compared with the control group, the thrombus in the caudal vein of the PHZ/AH treatment group was obviously increased, while the intensity of red blood cells (RBCs) in the heart decreased significantly, as shown in Figure 3(B1–B4). However, ASP, SAB, MA, LA, and LG could significantly reduce the chemical-induced thrombus in the caudal vein and recover the chemical-induced decrease in RBCs intensity in the heart.

**Figure 4:**
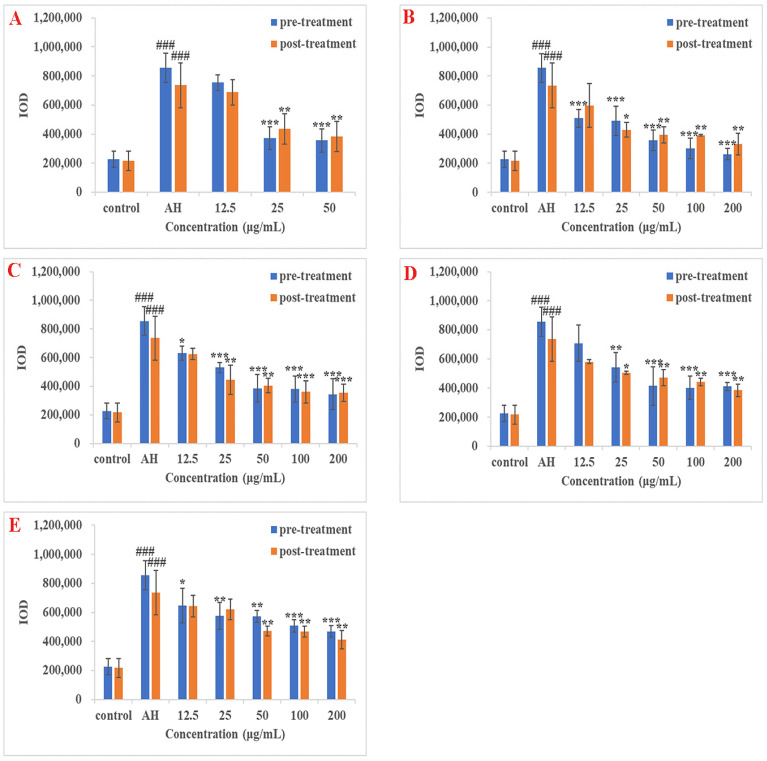
Result of the anti-thrombotic effect of DS components in AH-induced zebrafish thrombosis model. (**A**–**E**) indicated the results of ASP, SAB, MA, LA, and LG, respectively. The blue bar indicated the IOD value of the pre-treatment group, while the orange bar indicated the IOD value of the post-treatment group. ^###^ *p* < 0.001 versus control group; * *p* < 0.05, ** *p* < 0.01 and *** *p* < 0.001 versus AH group.

In AH-induced thrombus treatment groups, statistically significant anti-thrombotic effects in pre-treatment zebrafish groups were observed at 25 and 50 μg/mL (*p* < 0.001) for ASP; 12.5, 25, 50, 100, and 200 μg/mL (*p* < 0.001) for SAB; 12.5 μg/mL (*p* < 0.05), 25, 50, 100, and 200 μg/mL (*p* < 0.001) for MA; 25 μg/mL (*p* < 0.01), 50, 100, and 200 μg/mL (*p* < 0.001) for LA; 12.5 μg/mL (*p* < 0.05), 25 and 50 μg/mL (*p* < 0.01), 100 and 200 μg/mL (*p* < 0.001) for LG. These results are shown in Figure 4. The IC_50_ of ASP, SAB, MA, LA, and LG were calculated as 19.08, 12.22, 22.07, 31.12, and 62.75 μg/mL, respectively (Table 5). Statistically significant anti-thrombotic effects in post-treatment zebrafish groups were observed at 25 and 50 μg/mL (*p* < 0.01) for ASP; 25 μg/mL (*p* < 0.05), 50, 100, and 200 μg/mL (*p* < 0.01) for SAB; 25 and 50 μg/mL (*p* < 0.01), 100 and 200 μg/mL (*p* < 0.001) for MA; 25 μg/mL (*p* < 0.05), 50, 100, and 200 μg/mL (*p* < 0.01) for LA; 50, 100, and 200 μg/mL (*p* < 0.01) for LG (Figure 4). The IC_50_ of ASP, SAB, MA, LA, and LG were calculated as 27.22, 27.23, 31.03, 50.62, and 85.22 μg/mL, respectively (Table 5).

**Figure 5:**
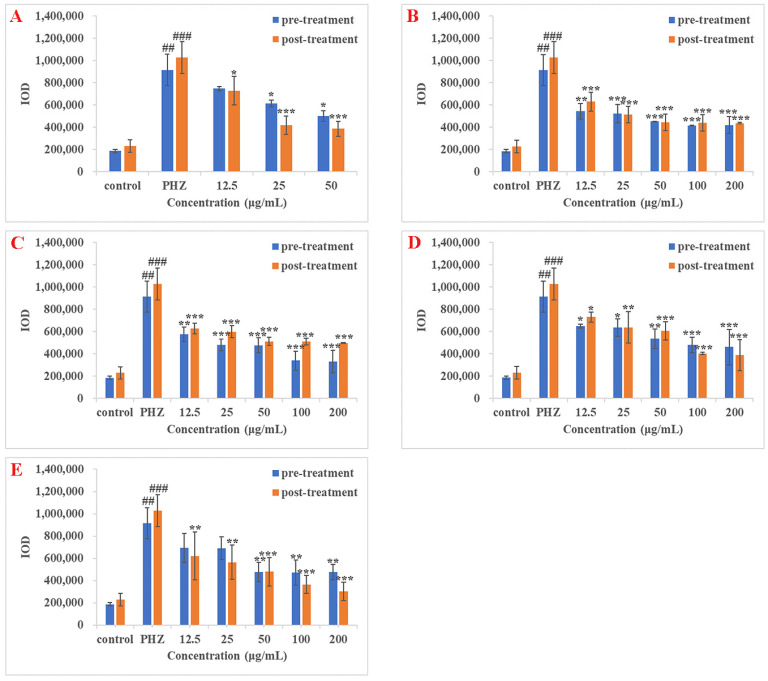
Result of the anti-thrombotic effect of DS components in PHZ-induced zebrafish thrombosis model. (**A**–**E**) indicated the results of ASP, SAB, MA, LA, and LG, respectively. The blue bar indicated the IOD value of the pre-treatment group, while the orange bar indicated the IOD value of the post-treatment group. ^##^ *p* < 0.01, ^###^ *p* < 0.001 versus control group; * *p* < 0.05, ** *p* < 0.01 and *** *p* < 0.001 versus PHZ group.

In the PHZ-induced thrombus treatment groups, statistically significant anti-thrombotic effects in pre-treatment zebrafish groups were observed at 25 and 50 μg/mL (*p* < 0.05) for ASP; 12.5 μg/mL (*p* < 0.01), 25, 50, 100, and 200 μg/mL (*p* < 0.001) for SAB; 12.5 μg/mL (*p* < 0.01), 25, 50, 100, and 200 μg/mL (*p* < 0.001) for MA; 12.5 and 25 μg/mL (*p* < 0.05), 50 μg/mL (*p* < 0.01), 100 and 200 μg/mL (*p* < 0.001) for LA; 50, 100 and 200 μg/mL (*p* < 0.01) for LG. These results are shown in Figure 5. The IC_50_ of ASP, SAB, MA, LA, and LG were calculated as 37.28, 11.28, 15.47, 50.59, and 55.34 μg/mL, respectively (Table 6). Statistically significant anti-thrombotic effects in post-treatment zebrafish groups were observed at 12.5 μg/mL (*p* < 0.05), 25 and 50 μg/mL (*p* < 0.001) for ASP; 12.5, 25, 50, 100, and 200 μg/mL (*p* < 0.001) for SAB; 12.5, 25, 50, 100, and 200 μg/mL (*p* < 0.001) for MA; 12.5 μg/mL (*p* < 0.05), 25 μg/mL (*p* < 0.01), 50, 100, and 200 μg/mL (*p* < 0.001) for LA; 12.5 and 25 μg/mL (*p* < 0.01), 50, 100, and 200 μg/mL (*p* < 0.001) for LG (Figure 5). The IC_50_ of ASP, SAB, MA, LA, and LG were 15.56, 8.11, 10.72, 27.95, and 14.01 μg/mL respectively (Table 6).

In conclusion, all of the four DS components, SAB, MA, LA, and LG exhibited a dose-dependent anti-thrombotic effect in AH/PHZ-induced zebrafish thrombosis model by both pre-treatment and post-treatment methods. Similar results were also observed in the ASP treatment group, which served as a positive control.

In recent years, polyphenols contained in fruits and vegetables had attracted considerable attention because of their benefits in human health, including the reduction of the risk of cardiovascular disease. SAB, MA, LA, and LG were all polyphenolic constitutions of DS and were proved to exert an anti-thrombotic effect in our study. Therefore, the result may suggest to a certain degree that the application of DS in TCM is coherent with the use of polyphenols in Western medicine for the treatment of cardiovascular disease.

## 3. Materials and Methods

### 3.1. Chemicals and Reagents

N-Phenylthiourea (PTU), phenylhydrazine (PHZ), (±)-epinephrine hydrochloride (AH), acetylsalicylic acid (aspirin, ASP), ethyl 3-aminobenzoate methanesulfonate (MS-222) and 3,3′-dimethoxybenzidine (O-dianisidine) were purchased from Shanghai Aladdin Biochemical Technology Co., Ltd. (Shanghai, China). Salvianolic acid B (SAB), rosmarinic acid (MA) and dimethyl sulfoxide (DMSO) were purchased from Shanghai Macklin Biochemical Co., Ltd. (Shanghai, China). Lithospermic acid (LA) was purchased from Chengdu Must Bio-Technology Co., Ltd. (Chengdu, China). Luteolin-7-O-β-D-glucoside (LG) was purchased from Shanghai Standard Technology Co., Ltd. (Shanghai, China). Gelatin was purchased from Beyotime Biotech Inc. Hydrogen peroxide (30%, H_2_O_2_) (AR) was purchased from Chengdu Kelong Chemical Co., Ltd. (Chengdu, China). Sodium acetate anhydrous (CH_3_COONa) (AR) was purchased from Tianjin Zhiyuan Chemical Reagent Co., Ltd. (Tianjin, China). Ethanol (AR) was purchased from Chongqing Chuandong Chemical (Group) Co., Ltd. (Chongqing, China). Other regular reagents for the daily maintenance of zebrafish system were purchased from Wuhan TianZhengYuan Biological Technology Co., Ltd. (Wuhan, China). DS compounds stock solutions were prepared in experimental water, DMSO, or absolute ethanol.

### 3.2. Maintenance and Husbandry of Zebrafish

Wild-type AB strain adult zebrafish were purchased from the Shanghai FishBio Co., Ltd. (Shanghai, China). Zebrafish were raised and kept in a flow-through farming system provided by Nanjing Eze Rinka Biotechnology Co., Ltd. (Nanjing, China) according to the standard procedure of China Zebrafish Resource Center. Zebrafish were maintained in light (under a 14:10 h light/dark cycle) and temperature (25–28 °C)-controlled conditions in water (pH 7.2–7.8 and conductivity 450–550 μs/cm) and fed twice daily with live brine shrimp and dry flake food. Embryos were obtained from spawning adults in a breeding chamber overnight with a sex ratio of 2:1 (male/female) according to the standard zebrafish breeding protocol.

### 3.3. Molecular Docking Studies

The structures of molecules of active ingredients in DS were obtained from TCMSP and saved to files in mol2 format. With Gasteiger–Huckel charge, Tripos force field, and the Powell energy gradient method, all molecules were optimized in SYBYL 2.0 software (TRIPOS, Inc., St. Louis, MO, USA). The Powell energy gradient method was used to optimize the molecules by setting the maximum number of times to 10,000 and the energy convergence level difference to 0.005 Kcal/mol [22,32] and the rest of the parameters were set to default values. According to this method, the lowest energy small molecule structure is regarded as the active conformation.

Target proteins related to thrombus were searched from the TTD and UniProt database. The crystal structure of the protein came from the PDB database. Prior to docking studies, proteins needed to be pre-treated, including residual repairs, energy minimization, the addition of hydrogen atoms, as well as the removal of co-crystallized ligands, water molecules, and metal ions from the complexes.

Molecular docking was carried out using a standard Surflex-Dock protocol in SYBYL 2.0 software. The docking pocket was formed with amino acid residues at 0.5 Å in the range of the original ligand molecule, and the remaining parameters were set as default. The original ligand was redocked into the binding pocket to verify the reliability of the docking method [22,32]. Then, compounds were docked into the binding pocket using the same parameters. Afterwards, by comparing the docking score with the original ligand, compounds with a higher value were screened out. 

### 3.4. Preparation of Solutions and Samples

First, 2.64 mg of AH was dissolved in 50 μL DMSO to prepare 240 mM stock solution, and 2.36 μL PHZ was dissolved in 997.64 μL absolute ethanol to prepare 24 mM stock solution. These stock solutions were further diluted to working solutions with 0.2 mΜ PTU-containing water, and the final concentrations of DMSO and absolute ethanol were kept below 0.1%. Thirty milligrams O-dianisidine and 41 mg CH_3_COONa were dissolved in 20 mL of absolute ethanol as the stock solution; and then, 128 μL 30% H_2_O_2_ and 20 mL of water were added to form O-dianisidine working solution. ASP, SAB, MA, LA, and LG were dissolved in DMSO to form the stock solutions; then, they were further diluted to working solutions (12.5, 25, 50, 100, and 200 μg/mL) with 0.2 mΜ PTU-containing water. 

### 3.5. AH/PHZ-Induced Zebrafish Thrombosis Model and DS Compounds Treatment

The zebrafish were raised in the water containing 0.2 mM PTU from 24 hpf (hours post fertilization) during all of the experiments. Two thrombus chemical inducers, AH or PHZ, were used respectively in zebrafish to induce the thrombosis model. Zebrafish embryos were applied to treatment in 24-well plates containing 15 fish per well; three parallel wells were set per treatment group. In the control group, zebrafish were treated with 0.1% DMSO or 0.1% absolute ethanol, respectively. In the AH/PHZ treatment group (AH/PHZ group), 15 μM AH or 1.5 μM PHZ were treated to 78 hpf zebrafish for 16 h or 24 h, respectively. In the ASP treatment group (ASP group), as a positive control, ASP (12.5, 25, 50, 100, and 200 μg/mL) was treated to zebrafish for 24 h either before (pre-treatment) or after (post-treatment) AH/PHZ treatment. In the DS compounds treatment group, SAB (12.5, 25, 50, 100, 200 μg/mL), MA (12.5, 25, 50, 100, 200 μg/mL), LA (12.5, 25, 50, 100, 200 μg/mL), and LG (12.5, 25, 50, 100, 200 μg/mL) were treated to zebrafish for 24 h either before (pre-treatment) or after (post-treatment) AH/PHZ treatment, respectively.

After DS compounds treatment, zebrafish were stained in the dark with O-dianisidine staining solution for 30 min. Then, the O-dianisidine working solution was abandoned, and the zebrafish were washed by DMSO three times. All of the images were taken at the same magnification, with an upright fluorescence microscopy imaging system. The software used to capture the images was cellSens Standard.

Zebrafish from each group were picked out for image capture of the heart and caudal vein RBCs, respectively. The images were quantitatively analyzed using Image-Pro Plus 6.0 according to IOD value. The anti-thrombotic effects of the test compounds were evaluated based on the formula below:

Efficacy(%) = [[IOD_(treatment group)_ − IOD_(AH/PHZ group)_]/[IOD_(control group)_ − IOD_(AH/PHZ group)_]] × 100% (1)

IC_50_ was calculated with GraphPad Prism 8.

### 3.6. Ethics Statements

All zebrafish experiments were conducted according to the guidelines of the Animal Ethics Committee of the School of Pharmacy and Bioengineering, Chongqing University of Technology.

### 3.7. Statistical Analysis

All data were expressed as the mean ± the standard error of mean (SEM) of three different experiments. Multiple group comparison was conducted by one-way analysis of variance (ANOVA) of IBM SPSS Statistics 19. A *p*-value of less than 0.05 was considered as statistically significant.

## 4. Conclusions

In this study, a rapid and effective method for anti-thrombotic effects screening in DS was established based on the zebrafish in vivo assay and computational studies of molecular docking. Molecular docking studies showed that SAB, MA, LA, and LG from DS exhibited the good binding affinity to the thrombosis-related proteins. Furthermore, SAB, MA, LA, and LG exhibited anti-thrombotic effects in the chemical-induced zebrafish thrombosis model. This study demonstrated a feasible approach for the screening of pharmacological effect of small molecules by combining a zebrafish in vivo model with molecular docking techniques. Meanwhile, our research also provided references for the further study of therapeutic effects of DS to thrombosis-related diseases.

## Figures and Tables

**Table 1:** The number of compounds in DS that could bind to thrombosis-related enzymes.

Number	Target Name	PDB ID	Number of Compounds
1	Antithrombin III (ATIII)	1R1L	160
2	Coagulation factor IIa (F2)	5AFY	143
3	von Willebrand factor (VWF)	4C2A	143
4	Coagulation factor XI (F11)	6TS4	110
5	Plasminogen activator inhibitor (PAI)	1JRR	100
6	Neuronal acetylcholine receptor beta-2 (CHRNB2)	6NCJ	88
7	DNA topoisomerase II (TOP2)	3QX3	73
8	Neuronal acetylcholine receptor alpha-2/alpha-3 (CHRNA2/A3)	4ZK4	50
9	Glycoprotein IIb/IIIa receptor (GPIIb/IIIa)	3T3M	43
10	Coagulation factor Xa (F10)	2JKH	40
11	P-selectin (SELP)	1G1S	38
12	Neuronal acetylcholine receptor alpha-2/alpha-3 (CHRNA2/A3)	5FJV	36
13	Neuronal acetylcholine receptor alpha-7 (CHRNA7)	5AFN	36
14	Tyrosine-protein kinase SYK (SYK)	4YJR	19
15	DNA topoisomerase II (TOP2)	1ZXN	16
16	P2Y purinoceptor 1 (P2RY1)	4XNV	5
17	Plasminogen activator inhibitor (PAI)	2HI9	4
18	Coagulation factor IX (F9)	5JB9	4
19	Carboxypeptidase B2 (CPB2)	4P10	3
20	Platelet glycoprotein Ib alpha (CD42b)	4CH2	3
21	Arachidonate 5-lipoxygenase (5-LOX)	3V99	2
22	Plasminogen activator inhibitor (PAI)	4G8R	2
23	Proteinase activated receptor 1 (F2R)	3VW7	1
24	P2Y purinoceptor 12 (P2RY12)	4PXZ	0
25	HMG-CoA reductase (HMGCR)	2R4F	0

**Table 2:** Compounds in DS that could combine with more than nine thrombosis-related binding targets.

Number	Mol ID	Molecule Name	Number of Targets
1	MOL000069	palmitic acid	15
2	MOL000131	EIC (linoleic acid)	15
3	MOL000675	oleic acid	15
4	MOL002229	HEPTACOSANE	15
5	MOL007060	lithospermic acid B	15
6	MOL007116	methylrosmarinate	15
7	MOL007129	potassium salvianolate d	15
8	MOL007139	salvianolic acid e	15
9	MOL000860	stearic acid	14
10	MOL004784	stenol	14
11	MOL007113	lithospermic acid	14
12	MOL007136	salvianolic acid a	14
13	MOL001219	satol	13
14	MOL004502	monomethyl lithospermate	13
15	MOL007083	Z-8-hexadecen-1-ol acetate	13
16	MOL007132	(2R)-3-(3,4-dihydroxyphenyl)-2-[(Z)-3-(3,4-dihydroxyphenyl)acryloyl]oxy-propionic acid	13
17	MOL000865	hexadecane	12
18	MOL000869	henicosane	12
19	MOL001394	oktadekan	12
20	MOL002376	pentacosane	12
21	MOL007039	henicosyl formate	12
22	MOL007055	9-methyl lithospermate b	12
23	MOL007103	dimetbyl lithosper-mate b	12
24	MOL007104	dimethyllithospermate	12
25	MOL007106	ethyl lithospermate	12
26	MOL007138	salvianolic acid d	12
27	MOL000128	nerylacetate	11
28	MOL002771	VIV	11
29	MOL007074	salvianolic acid b	11
30	MOL007135	salvianic acid c	11
31	MOL007137	salvianolic acid c	11
32	MOL007142	salvianolic acid j	11
33	MOL000009	luteolin-7-o-glucoside	10
34	MOL000054	L-	10
35	MOL000055	L-lysin	10
36	MOL000932	alpha-farnesene	10
37	MOL007044	3,7-dimethylocta-2,6-dien-1-yl formate	10

**Table 3:** Docking scores of SAB, RA, LA, and LG.

Molecular	1R1L	5AFY	4C2A	6TS4	3QX3	3T3M	2JKH	5AFN
Original ligand	2.18	3.61	3.93	3.88	3.31	6.36	6.22	6.71
SAB	4.12	**12.58**	**8.91**	**9.75**	4.50	7.38	6.60	7.12
MA	**6.60**	7.10	6.04	7.21	**5.01**	7.64	7.11	7.43
LA	2.78	9.14	8.40	7.57	4.19	**9.70**	**8.14**	**7.49**
LG	3.85	6.65	5.96	8.08	3.32	7.50	8.09	6.71

Numbers in bold format indicated the highest score of binding for each protein.

**Table 4:** Docking results of investigated small-molecule compounds with F11.

Compounds	Hydrogen Bonds	Other Amino Acid Residues
SAB	TYR59A, ARG37D, GLY193, ALA195, GLY216, ALA97, GLY218, CYS219, CYS40, LEU39	TRP215, LYS192, HIS57, LEU146, CYS191, ALA190
MA	LEU39, CYS40, ASP189, CYS219, LYS192	ALA195, GLY226, GLY216, ALA190, SER214, TRP215, CYS191
LA	CYS58, ARG37D, ALA195, GLY193, ASP194, ASP189, GLY218, CYS219	ALA97, HIS57, LYS192, TRP215, ALA190, GLY216
LG	ASP189, HIS57, GLU98, GLY193, ALA195, ASP194, TRP215, SER214, VAL227	GLY226, CYS219, ALA190, LYS192

**Table 5:** Anti-thrombotic effects of DS compounds on AH-induced zebrafish thrombosis.

DS Compounds	Concentrations (μg/mL)	Pre-Treatment Efficacy (%)	Pre-Treatment IC_50_ (μg/mL)	Post-Treatment Efficacy (%)	Post-Treatment IC_50_ (μg/mL)
ASP	12.5	16.06		9.23	
	25	77.01	19.08	57.59	27.22
	50	79.39		68.04	
SAB	12.5	54.90		26.68	
	25	57.71		58.95	
	50	79.05	12.22	65.63	27.23
	100	87.98		66.08	
	200	94.21		77.88	
MA	12.5	35.69		21.43	
	25	51.65		55.87	
	50	74.63	22.07	63.73	31.03
	100	75.34		72.39	
	200	81.38		73.49	
LA	12.5	23.52		29.87	
	25	49.61		44.55	
	50	69.93	31.12	50.66	50.62
	100	71.97		56.48	
	200	70.64		67.45	
LG	12.5	33.17		17.58	
	25	44.34		21.97	
	50	45.09	62.75	51.07	85.22
	100	55.21		51.29	
	200	61.34		62.57	

**Table 6:** Anti-thrombotic effects of DS compounds on PHZ-induced zebrafish thrombosis.

DS Compounds	Concentrations (μg/mL)	Pre-Treatment Efficacy (%)	Pre-Treatment IC_50_ (μg/mL)	Post-Treatment Efficacy (%)	Post-Treatment IC_50_ (μg/mL)
ASP	12.5	23.08		37.66	
	25	41.12	37.28	76.29	15.56
	50	56.94		80.27	
SAB	12.5	50.79		49.69	
	25	53.66		64.43	
	50	63.53	11.28	73.10	8.11
	100	68.42		73.40	
	200	67.79		74.00	
MA	12.5	46.48		50.15	
	25	59.87		53.82	
	50	60.10	15.47	64.78	10.72
	100	79.05		65.12	
	200	79.84		66.44	
LA	12.5	36.54		37.37	
	25	38.52		48.93	
	50	52.11	50.59	52.90	27.95
	100	59.69		78.32	
	200	62.43		80.13	
LG	12.5	30.33		50.89	
	25	30.77		58.06	
	50	60.26	55.34	68.82	14.01
	100	60.85		83.09	
	200	60.21		91.10	

## Data Availability

The data presented in this study are contained within the article.

## Sample Availability

Samples of all of the compounds are available from the authors.
